# Dissection of the cecal microbial community in chickens after *Eimeria tenella* infection

**DOI:** 10.1186/s13071-020-3897-6

**Published:** 2020-02-11

**Authors:** Hong-Liang Chen, Xin-Yu Zhao, Guang-Xun Zhao, Hai-Bin Huang, Hao-Rui Li, Chun-Wei Shi, Wen-Tao Yang, Yan-Long Jiang, Jian-Zhong Wang, Li-Ping Ye, Quan Zhao, Chun-Feng Wang, Gui-Lian Yang

**Affiliations:** College of Animal Science and Technology, Jilin Provincial Engineering Research Center of Animal Probiotics, Key Laboratory of Animal Production and Product Quality Safety of Ministry of Education, Jilin Agricultural University, Changchun, China

**Keywords:** *Eimeria tenella*, Chicken coccidiosis, Cecal microbiota, *16S* rRNA, Alternative therapeutics

## Abstract

**Background:**

*Eimeria* spp. are responsible for chicken coccidiosis which is the most important enteric protozoan disease resulting in tremendous economic losses in the poultry industry. Understanding the interaction between the avian cecal microbiota and coccidia is of interest in the development of alternative treatments that do not rely on chemotherapeutics and do not lead to drug resistance.

**Methods:**

We utilized *16S* rRNA gene sequencing to detect the dynamics of the cecal microbial community in AA broilers challenged with *Eimeria tenella*. Histopathological analysis of the cecum was also conducted.

**Results:**

We found that microbial shifts occur during the infection. *Lactobacillus*, *Faecalibacterium*, *Ruminococcaceae* UCG-013, *Romboutsia* and *Shuttleworthia* decreased in abundance. However, the opportunistic pathogens *Enterococcus* and *Streptococcus* increased in abundance over time in response to the infection.

**Conclusions:**

*Eimeria tenella* disrupts the integrity of the cecal microbiota and could promote the establishment and growth of potentially pathogenic bacteria. Defining bacterial populations affected by coccidial infection might help identify bacterial markers for intestinal disease as well as populations or species that could be beneficial in maintaining and restoring gut homeostasis during and after infection with *E. tenella*.

## Background

Avian coccidiosis is the most important protozoan disease for the poultry industry worldwide resulting in substantial economic losses [1]. Seven species of apicomplexan parasites belonging to the genus *Eimeria*, i.e. *E. tenella*, *E. necatrix*, *E. acervulina*, *E. maxima*, *E. brunetti*, *E. mitis* and *E. praecox*, are the causative agents of coccidiosis [2]. The invasion of *Eimeria* specifically damages the intestinal epithelial cells and tissues of the caecum and disrupts gut homeostasis. *Eimeria* infection increases intestinal colonization of pathogenic bacteria, such as *Clostridium perfringens* [3], *Salmonella enterica enterica* serovar Enteritidis [4] and *Campylobacter jejuni* [5] and causes large shifts in microbial community structure [6–9]. Currently chemotherapeutic drugs are extensively utilized to control and prevent coccidiosis, but this has led to an unavoidable increase in drug resistance and drug residues, which raises public health concerns for poultry meat [10]. With the emergence of anticoccidial and antibiotic drug resistance, alternative strategies are urgently required to prevent the disease.

The gut microbiota is an “invisible organ” that has been discovered to play pivotal roles in host health [11]. The intestinal microbiota contributes to harvesting nutrition and energy from the diet, reducing adhesion of enteric pathogens, stimulating the proliferation of the gut epithelium and promoting the development of the immune system [12]. A growing body of research has focused on well-defined bacteria that play a vital role in the modulation of intestinal homeostasis. Probiotics are viable, well-defined bacteria that contribute to the health and balance of the intestinal tract and have the potential to provide protection against chicken coccidiosis [13–17]. Probiotic strains were assessed *in vitro* for anticoccidial activity to inhibit *E. tenella* sporozoite invasion into Madin-Darby bovine kidney (MDBK) cells [18]. Therapeutic intervention of gut microbiota is considered as a promising alternative measure to control coccidiosis in the future, although little is known about the interactions between gut microbiota and enteric protozoans.

The majority of previous studies on the enteric microbiota of *Eimeria*-infected chickens were conducted *in vitro*, relying on bacterial cultivation and counting [19]. With the development of next-generation sequencing technology, high-throughput sequencing methods have provided a more direct way to analyze microbial taxa in comparison to culture-dependent methods, thus the microbial composition can be better characterized [20]. MacDonald et al. [21] reported that *E. tenella* infection induced significant changes in the abundance of some microbial taxa with notable differences detected between lesion score categories, and severe pathology was associated with an increase of *Enterobaceteriaceae* and a decrease of Bacillales and Lactobacillales. Huang et al. [22] also found that perturbation of the microbiota was observed both in Arbor Acres (AA) broilers and White Leghorn chickens by *16S* rRNA sequencing during the oocyst shedding period in response to *E. tenella* infection, *Clostridium* and *Escherichia* increased, *Lactobacillus* and *Faecalibacterium* decreased. In the present study, our aim was to determine the changes in bacterial populations belonging to the cecal lumen in AA broilers using *16S* rRNA gene sequencing, to explore the dynamics of the microbiota associated with the different phases of *E. tenella* infection within an intact life-cycle.

## Methods

### Chickens and parasites

One-day-old AA broiler chickens were obtained from ShuangYang Broiler Hatchery (Jilin, China) and reared under coccidia-free conditions in flame sterilized wire cages. Feed and water were supplied *ad libitum* and no antibiotics or anticoccidial drugs were used. Chickens were randomly divided into four groups of 10 birds per group.

The *E. tenella* Beijing strain was utilized in this study. The oocysts were sporulated and purified according to the methods described in our previous studies [23, 24].

### Experimental design

Forty AA broiler chickens with similar body weights were assigned equally into four groups: control group (C); merozoite reproduction group (M); gametocyte reproduction group (G); and oocyst shedding group (O). The control group was a non-infected group, and the other three groups were *E. tenella*-infected groups. At 21 days of age, chickens in infected groups were inoculated with 5 × 10^4^ *E. tenella* sporulated oocysts per chicken, while chickens in the control group were all sacrificed, the cecal contents of five birds were randomly selected and collected as fecal samples. At 105 h post-infection (hpi), all chickens in group M were sacrificed, and the cecal contents of five individuals were randomly selected and collected as fecal samples. Then at 144 hpi and 214 hpi, the stool samples of groups G and O were collected. All samples were stored at − 80 °C before DNA extraction and sequencing. These four groups represent four phases of an intact life-cycle of *E. tenella* infection.

### Histological evaluation

Cecal tissues from chickens from each group were excised, fixed in 10% formalin and embedded in paraffin wax. The tissues were dehydrated in a series of graded alcohols for staining and then sectioned. The sections were stained with hematoxylin and eosin (H&E) and examined microscopically.

### DNA extraction, PCR amplification and *16S* rRNA sequencing

The metagenomic DNA was extracted from the samples utilizing the E.Z.N.A.® Stool DNA Kit (Omega Bio-Tek, Norcross, GA, USA) according to the manufacturer’s instructions. The concentrations of the obtained DNA were determined by 1% agarose gel electrophoresis and spectrophotometry (optical density at 260 nm/280 nm ratio). Then the V3-V4 hypervariable region of the *16S* rRNA gene was amplified by PCR. A pair of universal primers (338F: 5ʹ-ACT CCT ACG GGA GGC AGC AG-3ʹ and 806R: 5ʹ-GAC TAC CVG GGT ATC TAA T-3ʹ) were used [25]. These primers contained a set of 8-nucleotide barcode sequences unique to each sample. The PCR program involved an initial denaturation step at 94 °C for 3 min, followed by 25 cycles of denaturation at 94 °C for 30 s, annealing at 50 °C for 30 s and extension at 72 °C for 60 s, with a final extension step at 72 °C for 7 min. PCRs were performed in triplicate 25 μl volumes containing 2.5 μl of 10× Pyrobest Buffer 2 μl (TaKaRa, Shiga, Japan), of 2.5 mM dNTPs, 1 μl of each primer (10 μM), 0.4 U of Pyrobest DNA polymerase (TaKaRa), and 15 ng of template DNA.

PCR products were run in an electrophoresis chamber on a 2% agarose gel and purified using the AxyPrep DNA Gel Extraction Kit (Axygen Biosciences, Union City, CA, USA) following the manufacturer’s instructions and quantified using QuantiFluor™-ST (Promega, Madison, Wisconsin, USA). Purified amplicons were used for library preparation and pyrosequencing. Sequencing libraries were generated using the NEBNext® Ultra™ DNA Library Prep Kit (New England Biolabs, Ipswich, MA, USA) following the manufacturer’s recommendations. Library quality was assessed and sequenced on an Illumina MiSeq platform PE300 platform (Illumina, Inc., CA, USA).

### Bioinformatics and sequencing data analysis

The original DNA fragments were merged into tags using Trimmomatic (version 0.36; http://www.usadellab.org/cms/?page=trimmomatic) and FLASH (Fast Length Adjustment of Short reads, version 1.2.11; https://ccb.jhu.edu/software/FLASH/) [26, 27]. Usearch (version 8.0.1623; https://www.drive5.com/usearch/) was applied to filter the chimeras and singletons of the raw sequencing data. Quality filtering of the raw tags was performed to generate high-quality clean tags according to Qiime (Quantitative Insights Into Microbial Ecology, v1.2.1; http://qiime.org/) [28]. Operational taxonomic units (OTUs) were clustered at 97% sequence similarity following the Uclust (version 1.2.22; https://drive5.com/usearch/manual/uclust_algo.html), and representative sequences of each cluster were used to assign taxonomy through annotation against the SILVA database. The alpha diversity of the samples, Chao 1 values, observed species and Shannon-Wiener indices were evaluated. Principal components analysis (PCA) of the OTUs in different groups was conducted using R version 3.5.1 Feather Spray (https://www.r-project.org/). Additionally, linear discriminant analysis (LDA) coupled with the effect size (LEfSe) algorithm (http://huttenhower.sph.harvard.edu/galaxy/) was conducted to identify the significant microbial differences among the groups [29]. The LDA score was calculated and a taxonomic cladogram was constructed to visualize the differences in microbial composition. A significance value of less than 0.05 and an LDA effect size of greater than 3 were used as thresholds for the LEfSe analysis. The raw reads were deposited into the NCBI Sequence Read Archive database (accession: SRP184532).

### Statistical analysis

Comparisons between experimental groups were carried out using ANOVA followed by Tukey’s honest significant differences (HSD) *post-hoc* test. All results are expressed as the mean ± standard error (SE). *P*-values of < 0.05 were considered significant.

## Results

### Histopathological analysis of the cecum

**Fig. 1:**
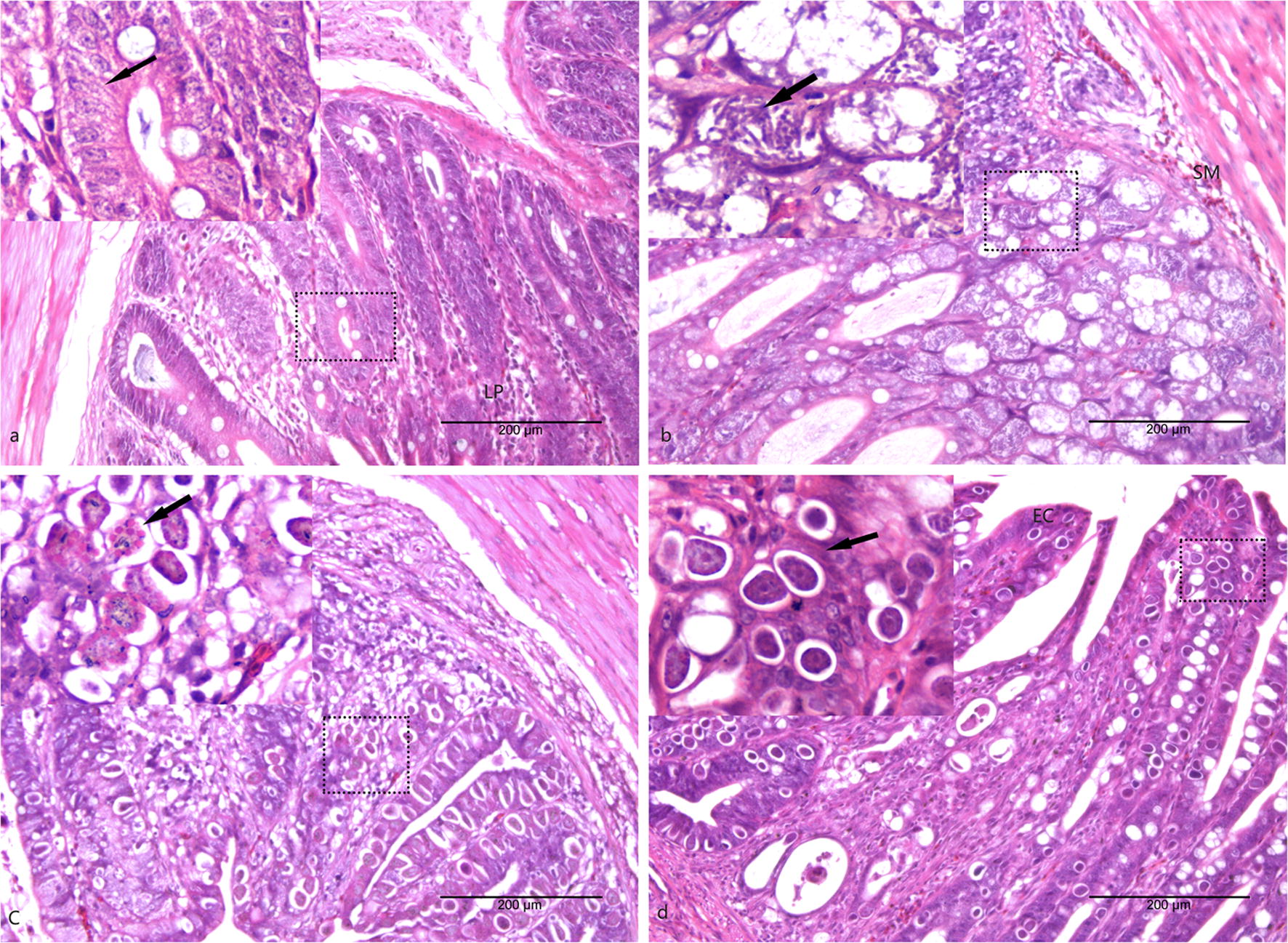
Histopathological images of the cecum in AA broiler chickens from each group. **a** Section of the cecum from the uninfected chickens. **b** Section of the cecum from the 105 hpi chickens in group M. **c** Section of the cecum from the 144 hpi chickens in group G; visible gametocytes are indicated by arrows. **d** Section of the cecum from the 214 hpi chickens in group O; visible oocysts are indicated by arrows. *Abbreviations*: EC, epithelial cell; LP, lamina propria; SM, submucosa. Magnifications: ×200 and ×400. *Scale-bars*: 200 μm

Microscopical examination showed that cecum glands were intact and no histopathological changes or necrosis were observed in the uninfected group (Fig. 1a). At 105 hpi, the structure of the cecum glands was indistinct and a large number of inflammatory cells were present in the submucosa (Fig. 1b). At 144 hpi, the intestinal glands and epithelial cells were invaded by coccidial gametocytes and oocysts. Inflammatory cells and villi blunting were also observed (Fig. 1c). At 214 hpi, several oocysts were observed in cecal glands and intestinal epithelial cells (Fig. 1d).

### Alpha and beta diversity of cecal microbial constitution after *E. tenella* infection

**Fig. 2:**
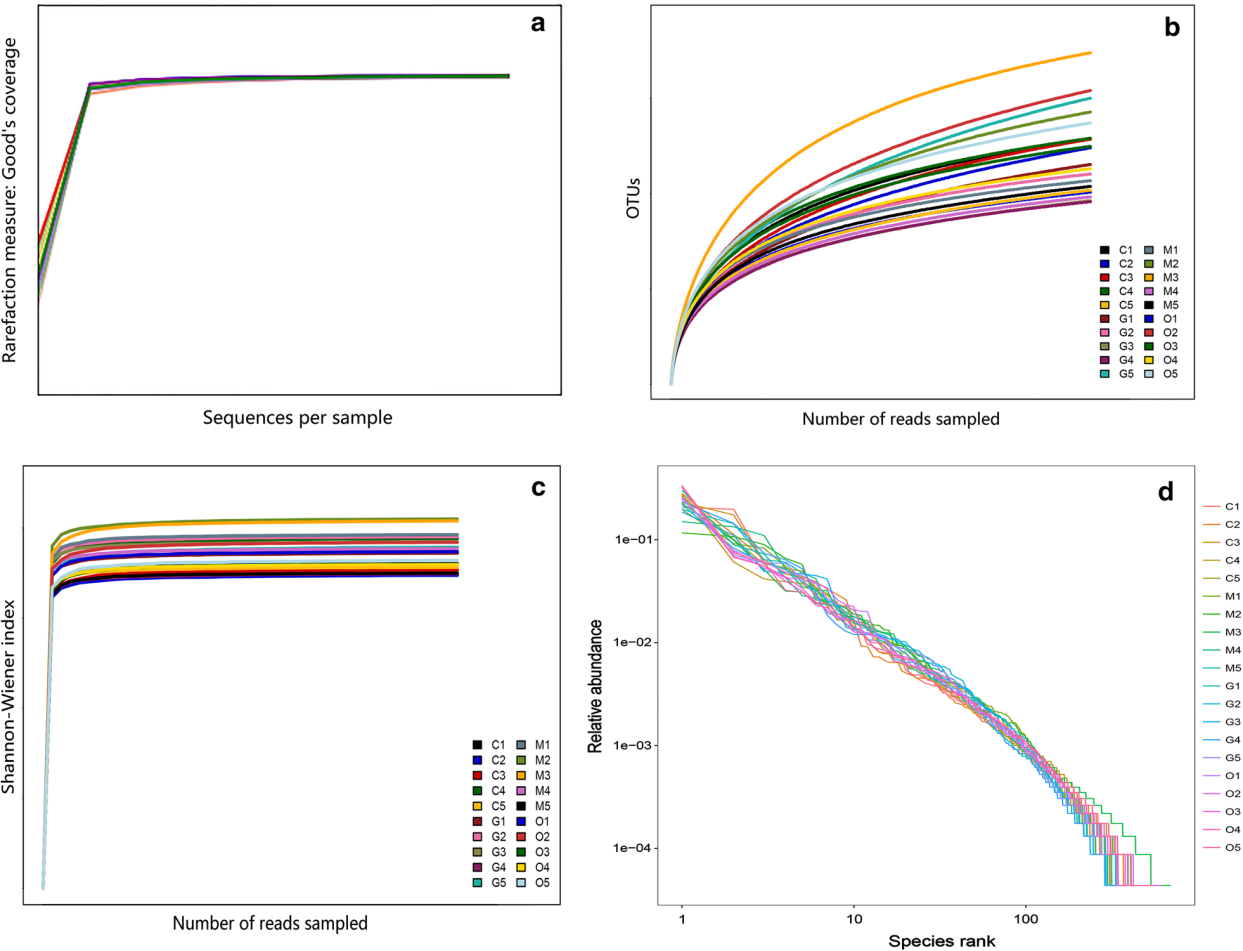
Curves for the OTUs obtained from 20 samples. **a** Good’s coverage analysis of sequencing data. **b** Rarefaction curves. **c** Shannon-Wiener curves. **d** Species accumulation curves. *Abbreviations*: C, samples from the control group; M, samples from the merozoite reproduction group; G, samples from the gametocyte reproduction group; O, samples from the oocyst shedding group

The *16S* rRNA gene-based sequencing produced millions of raw reads. After assembly and filtration, a total of 1,053,731 sequences were obtained through MiSeq sequencing analysis from 20 samples, and the average length of the sequences was 419.16 bp. The Goodʼs coverage index was greater than 99%. In total, 1125 OTUs were observed in the four experimental groups. Good’s coverage, the rarefaction, Shannon-Wiener, and OTU rank-abundance curves of all samples indicated that there was sufficient data sampling and adequate sequencing depth, and the database of *16S* rRNA gene sequences almost completely covered all microbial communities (Fig. 2).

**Fig. 3:**
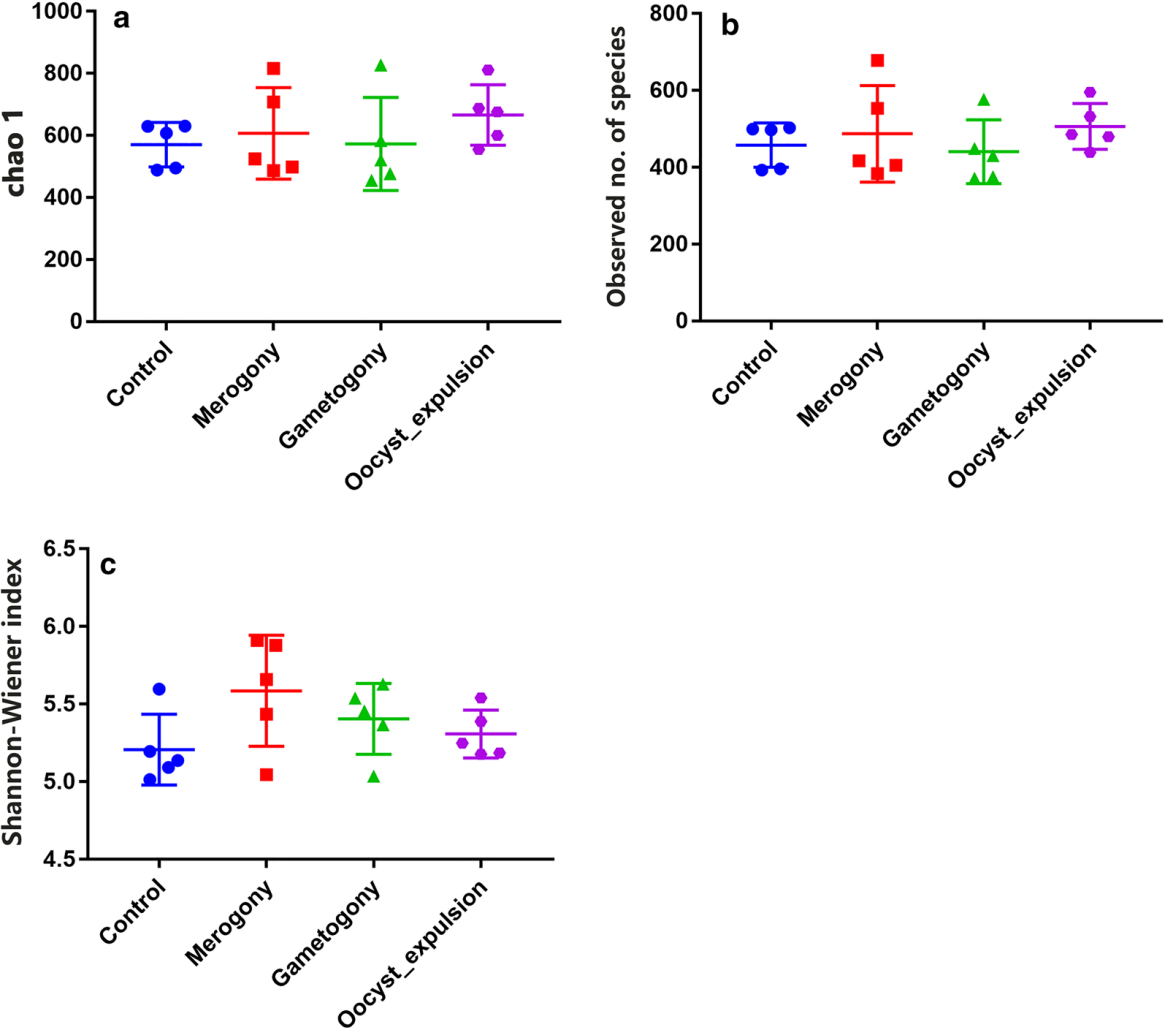
Analysis of alpha-diversity in the four experimental groups. Chao1 (**a**) and observed number of species (**b**) were used as richness estimators. Shannon-Wiener index (**c**) was used as a diversity estimator

The Chao 1 and observed species indices that estimate microbial richness, and the Shannon-Wiener index which measures species biodiversity, were calculated to evaluate the alpha diversity. Consideration of alpha diversity within the sequence datasets using the number of obtained OTUs, Chao 1, observed species and Shannon indices, showed no significant variation associated with *E. tenella* infection. There was no significant difference observed in the cecal microbiota alpha diversity between the control group and the infected groups. However, compared with the microbial diversity of the control group, the microbial biodiversity of the other groups increased after *E. tenella* infection. According to the Chao 1 index and observed species, we found that *E. tenella* infection led to an increase at the time point of 105 hpi in group M, then a slight decrease at the time point of 144 hpi. In group G, at the same time points, the Shannon-Wiener index indicated a similar trend. The microbial richness index in group O was the largest, and the Shannon-Wiener index of this group was lower than that of the other infected groups, but still higher than that of the control group. In conclusion, microbial shifts occurred in the cecum after *E. tenella* invasion. With the occurrence and development of invasion, microbiota was influenced at distinct time points (Fig. 3).

**Fig. 4:**
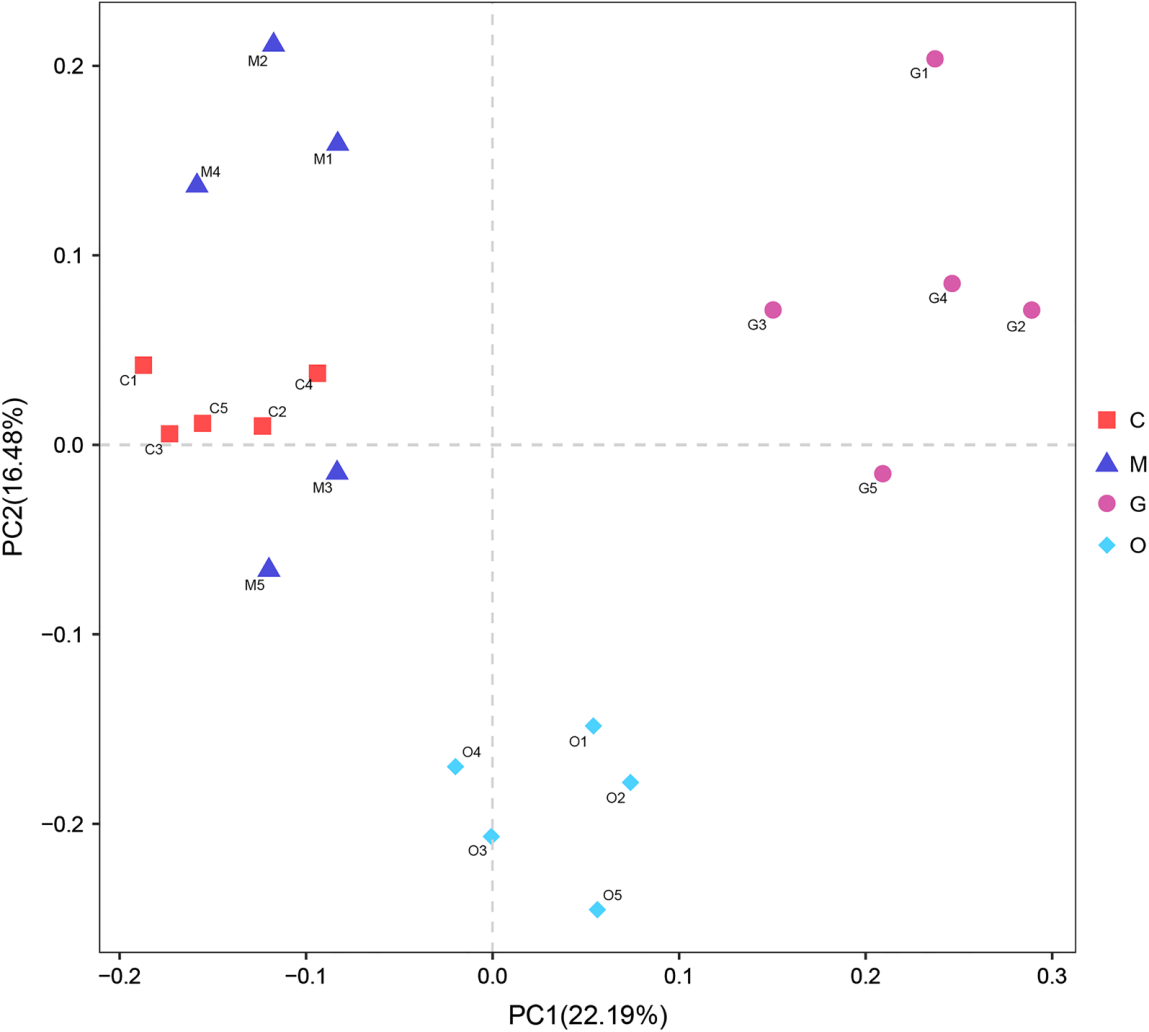
Principal coordinates analysis of the structure of the gut microbiota. *Abbreviations*: C, samples from the control group; M, samples from the merozoite reproduction group; G, samples from the gametocyte reproduction group; O, samples from the oocyst shedding group

Principal coordinates analysis (PCoA) and NMDS ordination plots indicated that there was a small variation in the cecal constitution between group C and group M. The cecal microbial composition in groups G and O had increased similarity, which means little variation occurred from 144 hpi to 214 hpi. Furthermore, an alteration was observed in the microbial structure between group M and group G, from 105 hpi to 144 hpi. In combination with the alpha diversity results, the observed species and Shannon-Wiener indices showed a clear decline from 105 hpi to 144 hpi, and the experimental data were consistent. Overall, the impacts of coccidial infection on the microbial community structure were roughly similar in agreement with the alpha diversity results in AA broiler chickens (Fig. 4).

### Bacterial taxa in the cecum after *E. tenella* infection

**Fig. 5:**
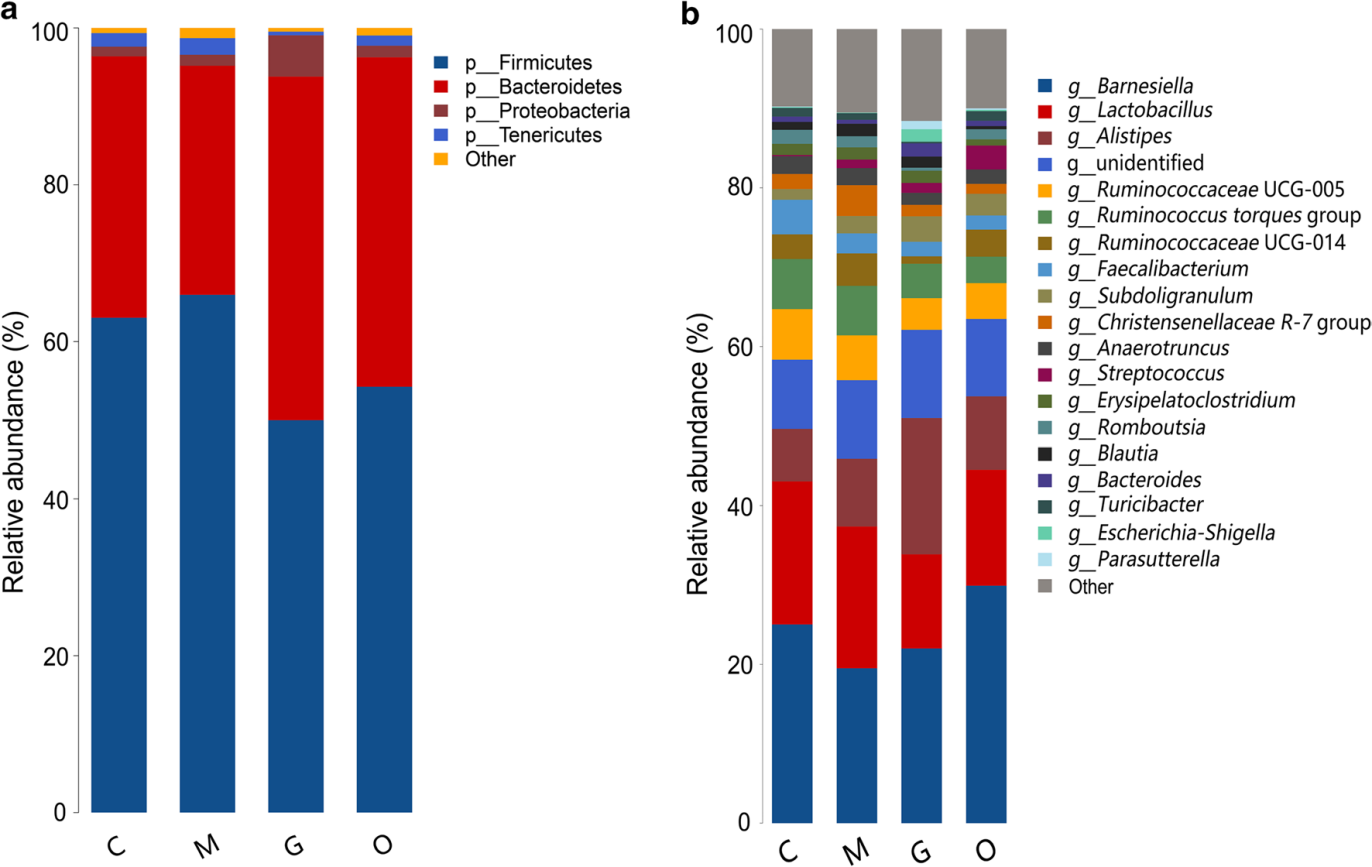
The relative abundances of the cecal microbiota at the phylum (**a**) and genus (**b**) levels. The relative abundances of the gut bacteria presented here were calculated by averaging the data obtained from the five replicates within each group. *Abbreviations*: C, control group; M, merozoite reproduction group; G, gametocyte reproduction group; O, oocyst shedding group

To elucidate the effect of *E. tenella* infection on the composition of the cecal microbiota, we analyzed the bacteria at the phylum and genus levels to characterize the dynamics of microbial taxonomic distribution. At the phylum level, Firmicutes, Bacteroidetes, Tenericutes and Proteobacteria dominated the cecal microbial community in all four groups. In group C, the relative abundance of Firmicutes, Bacteroidetes, Tenericutes and Proteobacteria was 63.06%, 33.32%, 1.72% and 1.24%, respectively. In group M, the relative abundance of Firmicutes and Tenericutes increased to 65.98% and 2.11%, respectively, while the abundance of Bacteroidetes and Proteobacteria decreased to 29.20% and 1.14%, respectively. In addition, we also observed Firmicutes (49.98%), Bacteroidetes (43.82%), Tenericutes (0.46%) and Proteobacteria (5.26%) in Group G, and Firmicutes (54.25%), Bacteroidetes (42.01%), Tenericutes (1.29%) and Proteobacteria (1.47%) in Group O. The results suggest that the dominant microbes in group C and in group M had increased similarity, and group G and group R had increased similarity at the phylum level (Fig. 5).

**Fig. 6:**
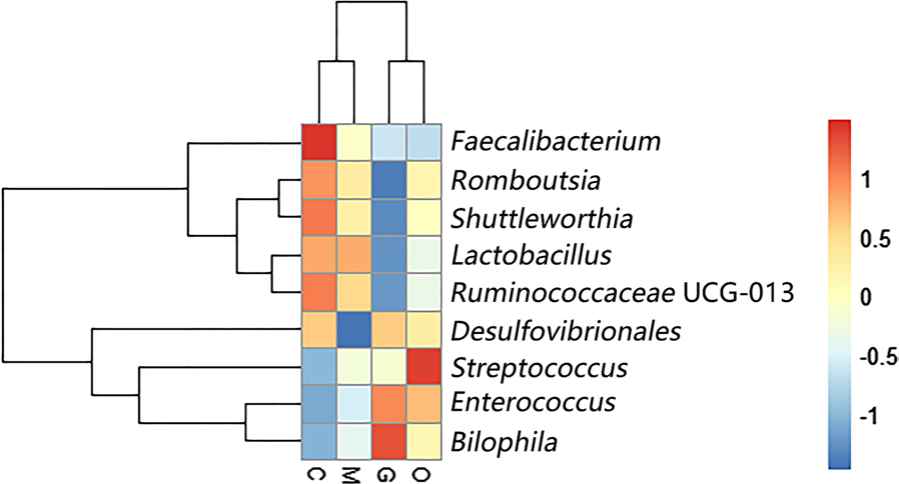
Heatmap plot depicting the relative abundance of each bacterial genus. *Abbreviations*: C, control group; M, merozoite reproduction group; G, gametocyte reproduction group; O, oocyst shedding group

We further compared the bacterial composition in the cecum at the genus level. A heatmap was also constructed based on the abundance profiles of the genera. *Lactobacillus* and *Faecalibacterium* belonging to the phylum Firmicutes, were important commensal microbiota. The relative abundance of *Lactobacillus* accounted for 18.02%, 17.86%, 11.87% and 14.59% of the population in groups C, M, G and O, respectively, which showed a clear decline during the *E. tenella* infection period. The relative abundance of *Faecalibacterium* was 4.36%, 2.51%, 1.83% and 1.79% in groups C, M, G and O, respectively. A linear decrease was also observed. *Ruminococcaceae* UCG-013 is a member of the Order Clostridiales, within the phylum Firmicutes. The relative abundance of *Ruminococcaceae* UCG-013 was outside of the abundance of the top 20 genera of all the samples. The heatmap showed that relative abundance of *Ruminococcaceae* UCG-013 decreased steadily with the development of the *E. tenella* infection (Fig. 6).

The heatmap also revealed that opportunistic pathogenic bacteria increased in relative abundance in the infected groups compared with the control group. Given the experimental data at different infection time points, we found that *Enterococcus* and *Streptococcus*, which both belong to the phylum Firmicutes, showed high abundance during *E. tenella* infection over time. The experimental results indicated that *E. tenella* infection impacts the microbiota composition in different phases of infection.

**Fig. 7:**
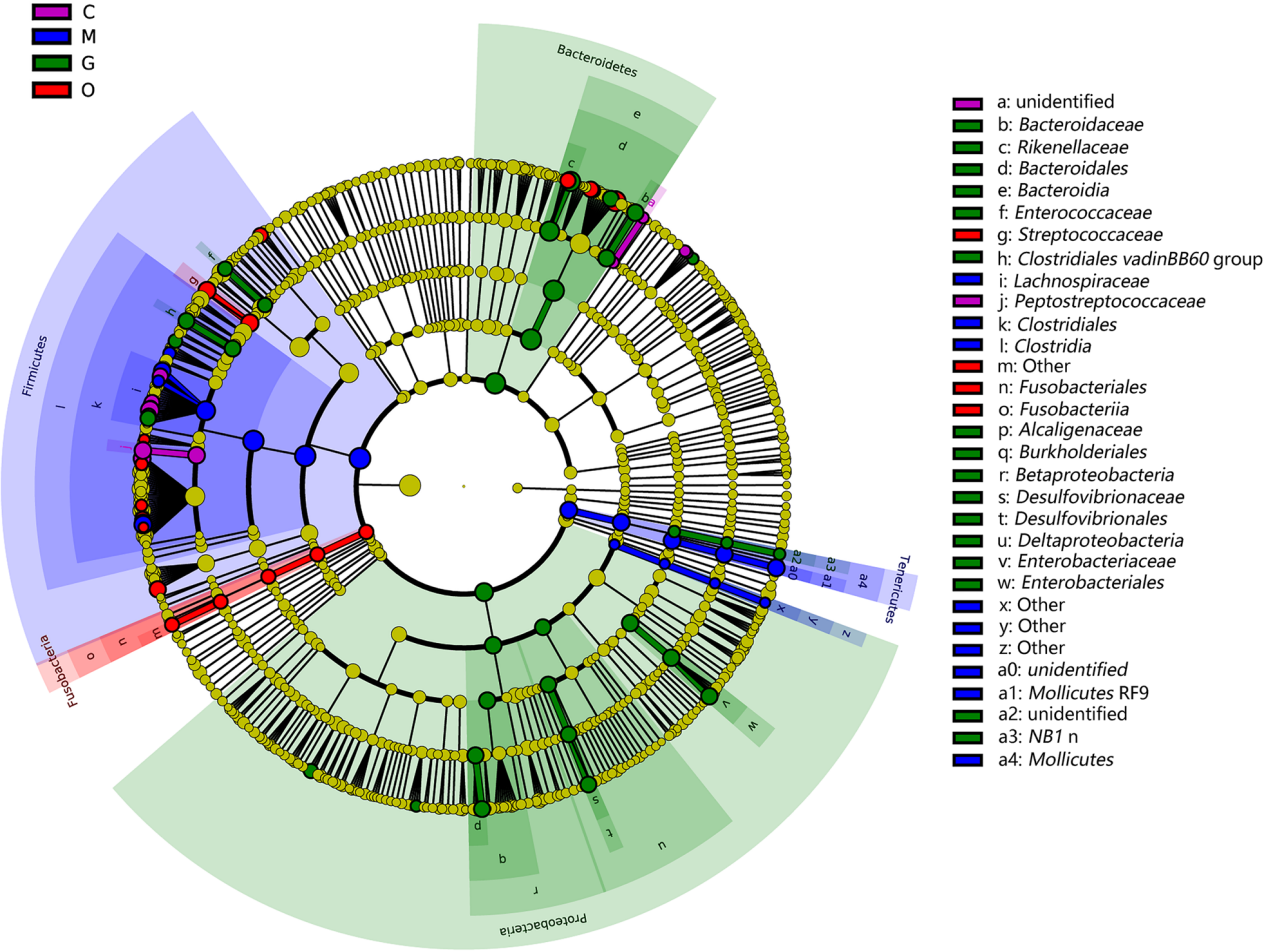
Cladogram of the LEfSe analysis of the gut microbiota in different groups. The microbial compositions were compared at different evolutionary levels

**Fig. 8:**
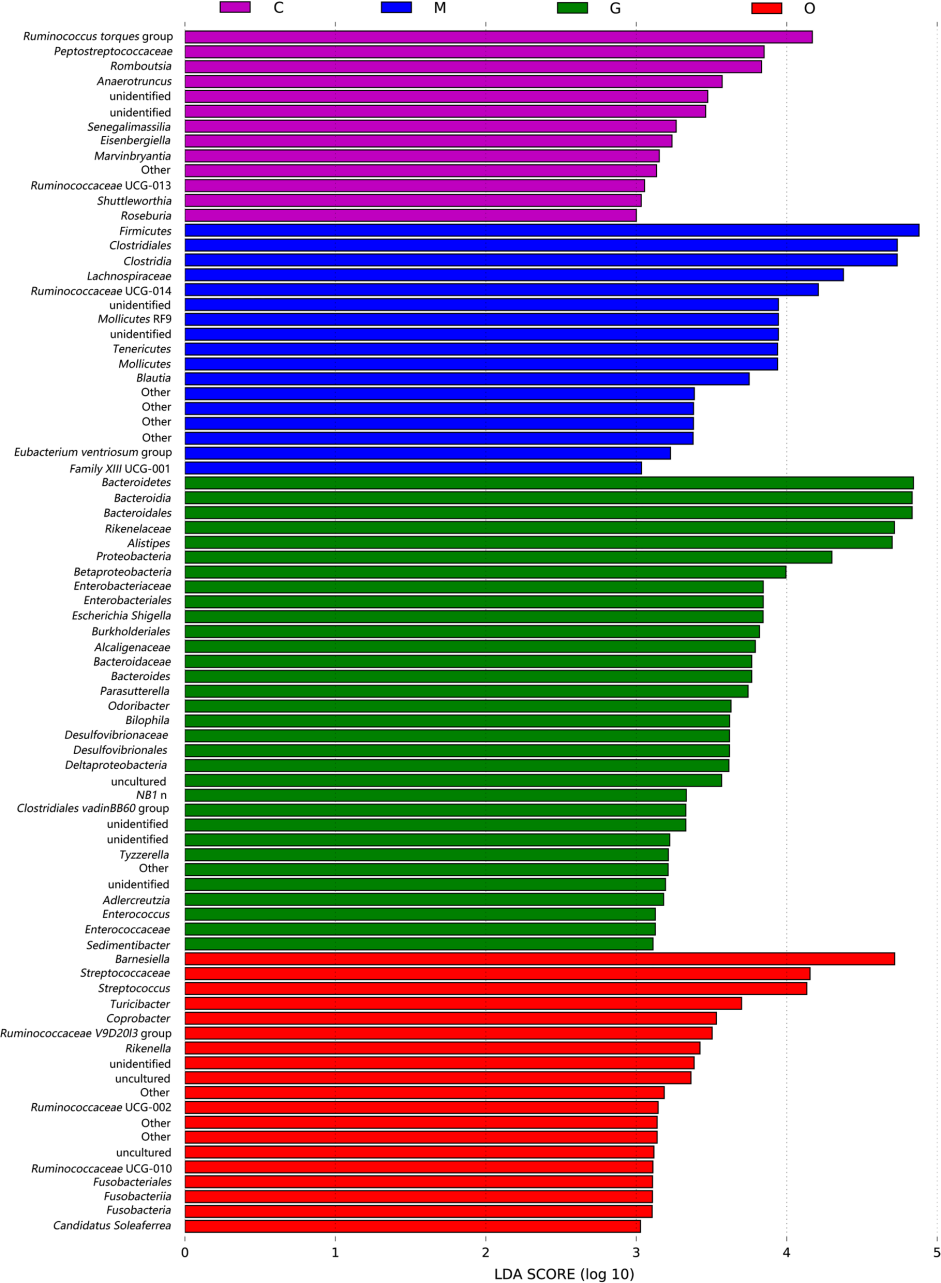
LDA scores obtained from the LEfSe analysis of the gut microbiota in different groups. An LDA effect size of greater than 3 was used as a threshold for the LEfSe analysis. *Abbreviations*: C, control group; M, merozoite reproduction group; G, gametocyte reproduction group; O, oocyst shedding group

**Fig. 9:**
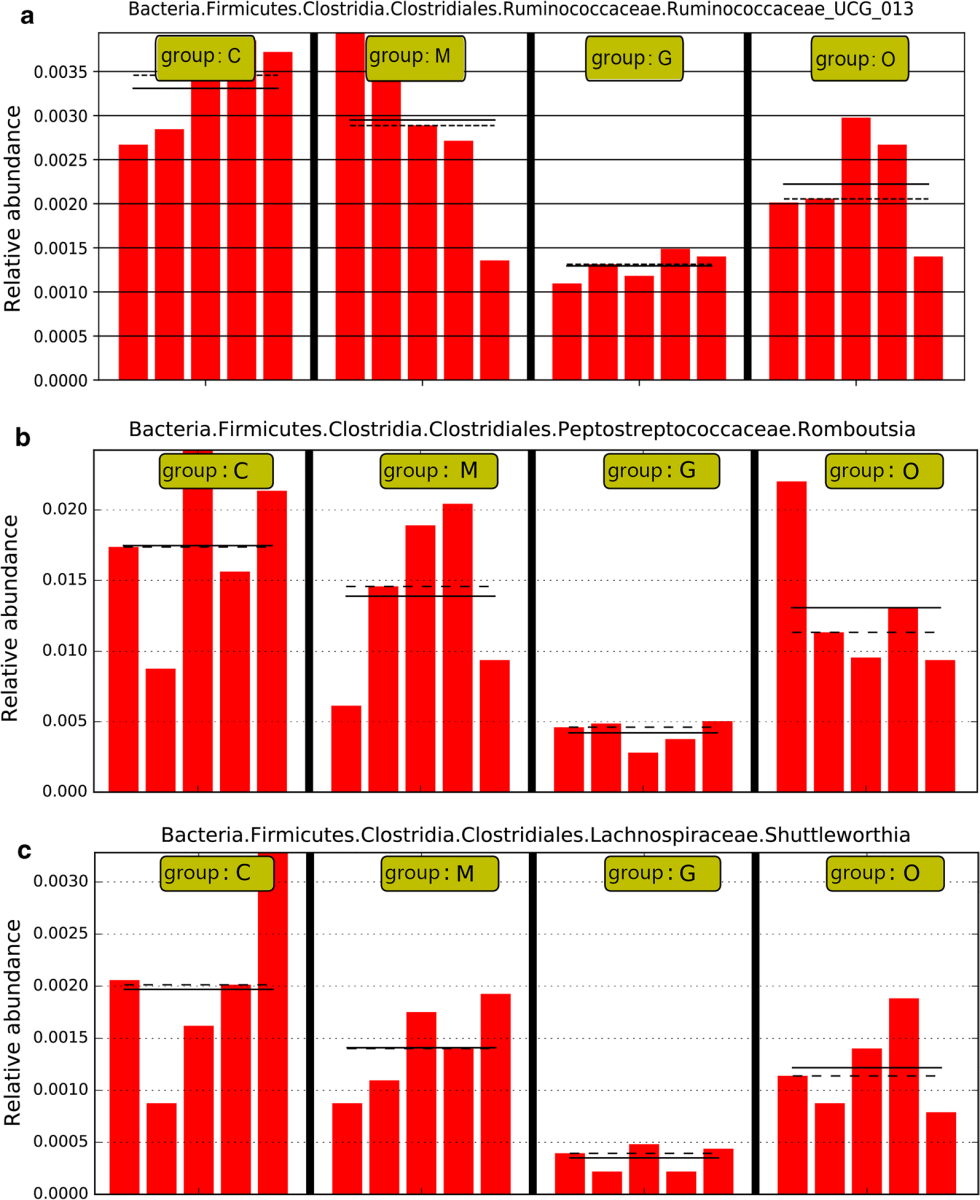
*Eimeria tenella* infection significantly decreased the abundances of *Ruminococcaceae* UCG-013 spp. (**a**), *Romboutsia* spp. (**b**) and *Shuttleworthina* spp. (**c**). *Abbreviations*: C, control group; M, merozoite reproduction group; G, gametocyte reproduction group; O, oocyst shedding group. The solid lines represent the mean values of relative abundance and the dotted lines represent the median values

**Fig. 10:**
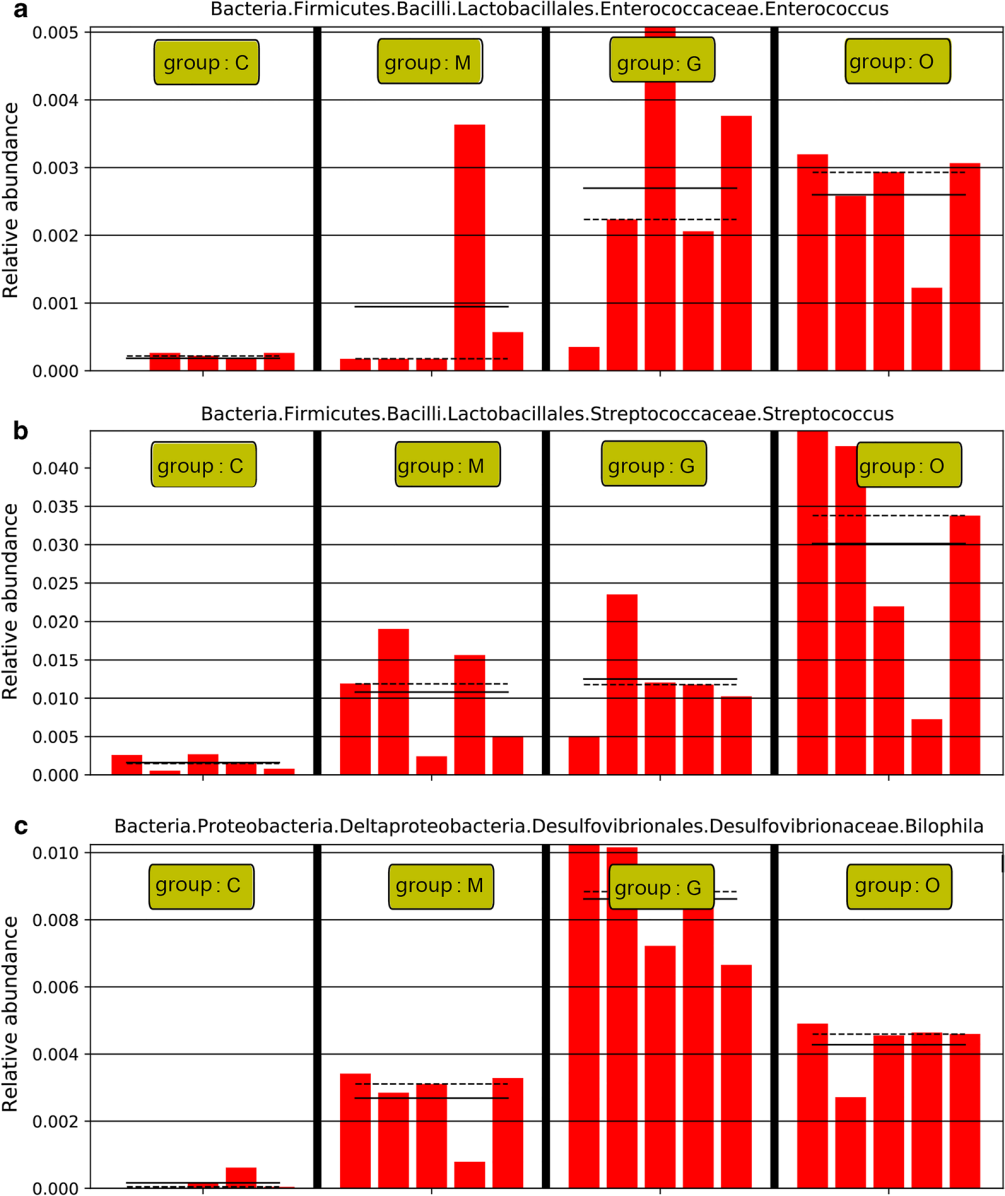
*Eimeria tenella* infection significantly increased the abundance of *Enterococcus* spp. (**a**), *Streptococcus* spp. (**b**) and *Bisophila* spp. (**c**). *Abbreviations*: C, control group; M, merozoite reproduction group; G, gametocyte reproduction group; O, oocyst shedding group. The solid lines represent the mean values of relative abundance and the dotted lines represent the median values

**Fig. 11:**
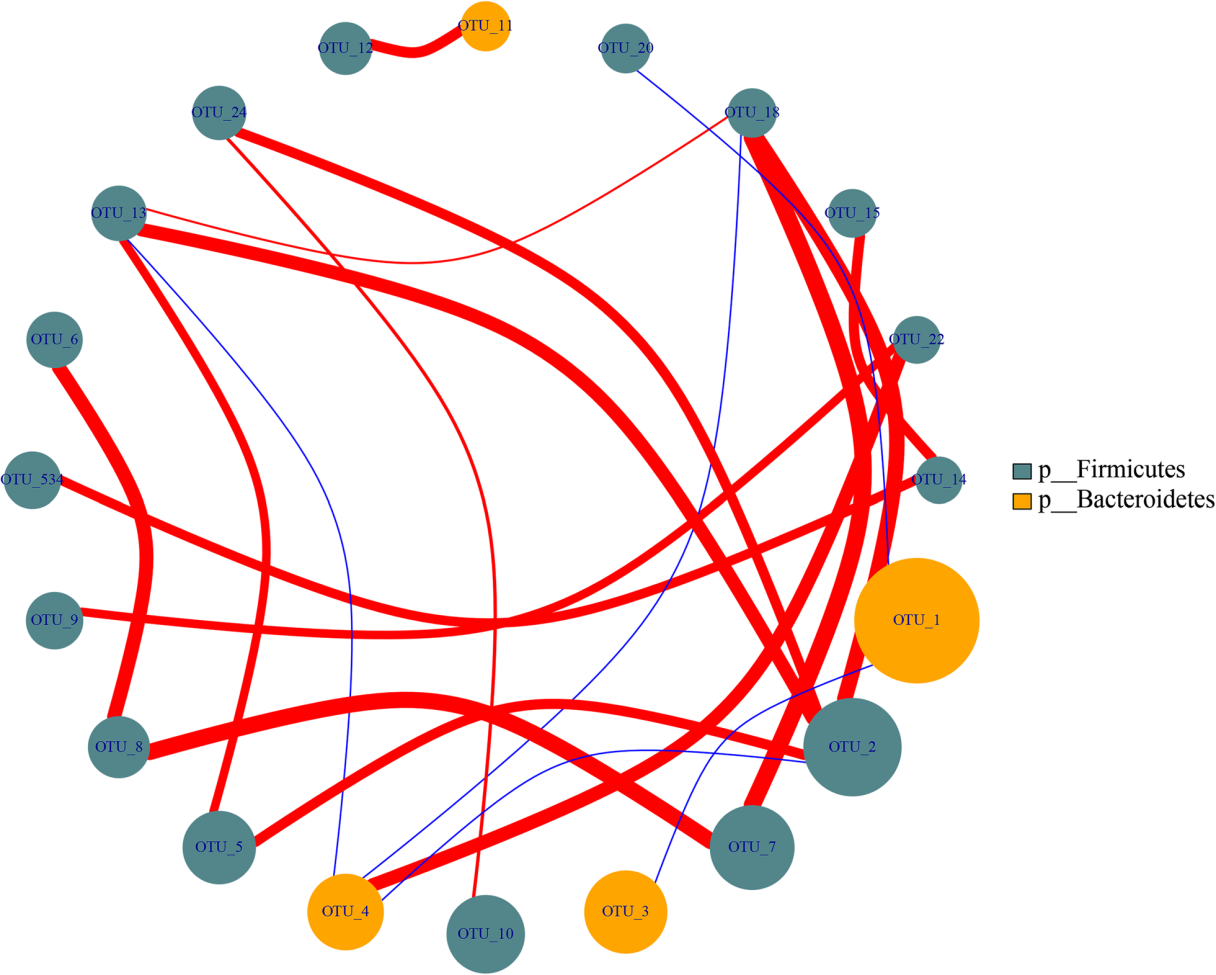
Co-occurrence network diagram of Firmicutes and Bacteroidetes

To fully understand the influence of *E. tenella* infection on gut microbiota, we performed a LEfSe analysis. The taxonomic cladogram and LDA score obtained from the LEfSe analysis confirmed and enabled the visualization of the impacts of infection (Figs. 7, 8). The LEfSe analysis showed a significant decline in the abundances of the potentially beneficial bacteria *Ruminococcaceae* UCG-013 between the infected groups and the control group. Specifically, compared with the uninfected chickens, the amount of *Ruminococcaceae* UCG-013 was decreased by approximately 2-fold at 144 hpi in the infected chickens. The populations of *Ruminococcaceae* UCG-013 showed a mild increase at 214 hpi, which suggests the possibility of the recovery of the gut environment. We also found that the populations of *Enterococcus* spp., *Streptococcus* spp. and *Bilophila* spp. were increased over time in response to coccidia invasion. Co-occurrence network diagram of Firmicutes and Bacteroidetes was also conducted to show the relationship between some important members belonging to the top two dominant phylum (Figs. 9, 10, 11).

## Discussion

Understanding the intestinal microbial composition and structure in chickens throughout the life-cycle of coccidial infection may help identify correlations between microbiota alterations and protozoan invasion over time, reveal potential biomarkers and may lead to the development of novel practical treatment methods [30]. In this study, we dissected the gut microbiota composition and structure in AA chickens at four time points during *E. tenella* infection utilizing *16S* rRNA gene sequencing. We found that commensal bacteria, such as *Lactobacillus*, *Faecalibacterium*, *Ruminococcaceae* UCG-013, *Romboutsia* and *Shuttleworthia*, declined in abundance after infection, whereas *Enterococcus* and *Streptococcus* were enriched in abundance in response to the infection. This finding is consistent with the microbiota constitution at the phylum level. All of these decreased genera mentioned above belong to the phylum Firmicutes. In the merozoite reproduction period of infection, at the 105 hpi time point, the levels of Firmicutes barely changed, from 63.06% to 65.98%. However, in the gametocyte reproduction period, at the 144 hpi time point, the abundance levels of Firmicutes markedly declined from 65.98% to 49.98%, which indicates the possibility of a decrease in the abundance of beneficial bacteria, and an increase in the emergence of opportunistic pathogenic bacteria. Our experimental results suggest that the late phase of the life-cycle of *E. tenella* infection in AA broilers impacted the homeostasis of the gut microbiota. The deficiency of defined resident microbiota may contribute to the pathogenicity of cecal coccidiosis.

Consistent with previous research, our study found that Firmicutes, Bacteroidetes, Tenericutes and Proteobacteria are the most common phyla in the chicken cecum. Firmicutes are the predominant phylum with the highest abundance [31]. The predominant phylum was mainly represented by *Lachnospiraceae*, *Ruminococcaceae*, *Lactobacillaceae*, *Peptostreptococcaceae* and *Clostridiaceae* [32]. Some members of the phylum Firmicutes can inhibit the growth of opportunistic pathogens and some are known to be involved in the degradation of complex carbohydrates [33]. The abundance levels of Firmicutes were decreased in the late phase of infection in our study. Moreover, the genera *Lactobacillus* (*Lactobacillaceae*), *Faecalibacterium* (*Clostridiaceae*), *Shuttleworthia* (*Lachnospiraceae*), *Ruminococcaceae* UCG-013 (*Ruminococcaceae*) and *Romboutsia* (*Peptostreptococcaceae*) showed a coccidian-associated reduction in the life-cycle of asexual (105 hpi) and sexual (144 hpi) replication processes, with a particularly clear reduction in the sexual reproduction period. *Lactobacillus*, which is a beneficial commensal for humans and animals, has been studied and used in medicine and the food industry for years. It has been proven that the enrichment of *Lactobacillus* could generally improve the gastrointestinal tract environment, protect the gut from pathogens and promote intestinal mucosal immunity and energy extraction in the host [34, 35]. *Faecalibacterium prausnitzii*, which is the only species of *Faecalibacterium*, is regarded as a potentially beneficial microbe because it has been shown to have anti-inflammatory properties in humans and murine models [36, 37]. In addition, as a saccharolytic, butyrate-producing bacterium, *Faecalibacterium* is speculated to express enzymes favoring the production of butyrate to regulate the immune system, to reduce chronic inflammation, and also alleviate the pathogenicity of *E. tenella* infection [38, 39]. *Ruminococcaceae* are common intestinal microbiota that degrade complex carbohydrates [40]. *Ruminococcaceae* have carbohydrate-active enzymes, sugar transport mechanisms, and metabolic pathways for the degradation of complex plant materials [41]. A reduction in *Ruminococcaceae* UCG-013 has been related to a disrupted carbon metabolism, which means body weight loss in chickens after *E. tenella* infection [42]. *Romboutsia* is a recently described bacterial genus that is usually associated with the health status of the gastrointestinal tract. The drastic reduction of this particular genus in intestinal mucosa may represent a potential microbial indicator of a disease condition. *Romboutsia* may play a crucial role in maintaining the health status of the host and could be a very valuable candidate biomarker of intestinal dysbiosis [43, 44]. *Shuttleworthia* has been demonstrated to have a relationship with carbohydrate and lipid metabolic pathways and thus contribute to weight gain and growth performance in broiler chickens [45]. However, opportunistic pathogenic bacteria of the genera *Enterococcus* and *Streptococcus* showed consistent increases in the infected AA chickens over time. *Bisophila*, which is a common non-pathogenic resident in the cecum of chickens, appears to be a consistent member of the anaerobic microbiota and may play a role in avian malabsorption syndromes [46].

LEfSe analysis indicated that *Ruminococcaceae* UCG-013, *Shuttleworthia* and *Romboutsia* may act as candidate biomarkers of coccidiosis. *Ruminococcaceae* UCG-013 and *Shuttleworthia* both play a role in regulating the carbohydrate metabolic pathway, which may impact host digestion. In addition, from the co-occurrence network diagram, positive correlations were found to exist between *Romboutsia* (OTU13) and *Lactobacillus* (OTU2), *Faecalibacterium* (OTU534) and *Ruminococcaceae* UCG-013. Our findings highlight the possibility of using butyrate-producing bacteria such as *Faecalibacterium*, probiotics such as *Lactobacillus*, carbohydrate-degrading bacteria such as *Ruminococcaceae* UCG-013 and *Shuttleworthia*, to reorganize gut microbiota to control coccidia infection.

The life-cycle of *E. tenella* includes asexual and sexual cycles. The asexual cycle consists of sporozoite and merozoite reproduction. The sexual cycle comprises microgamete reproduction. All reproductive processes occur inside the cecal epithelial cells [47]. In groups C and M, at the early phase time point of 105 hpi, we found that the microbiota composition and structure were similar in the early phase, in agreement with the earlier study of *Cryptosporidium* infection, which is related to *Eimeria* [48]. In groups M and G, from 105 hpi to 144 hpi, beneficial bacteria decreased, and conditional pathogenic bacteria increased. Huang et al. [22] found similar performance both in the infected AA broiler and White Leghorn chickens at 120 hpi, where the relative abundance of *Lactobacillus* and *Faecalibacterium* decreased, and the relative abundance of the pathobionts *Clostridium, Lysinibacillus* and *Escherichia* increased. In our study the microbiota was greatly impacted, but no significant variation in alpha diversity was observed between the control group and the infected groups. From 105 hpi to 144 hpi, *E. tenella* infection causes serious intestinal epithelial injuries that negatively impact the colonization and growth of resident bacteria, thus leading to decreased richness and diversity of cecal microbiota and increased risk of secondary infection [49]. MacDonald et al. [21] also reported that *E. tenella* infection induced no significant changes in the diversity of taxa in cecal microbial constitution, while the relative abundances of some genera such as *Lactobacillus*, *Bifidobacterium* and *Clostridium* changed significantly between all uninfected samples and all infected samples. These authors also described some interesting findings that the birds which remained asymptomatic after *E. tenella* infection had increased levels of *Lactobacillus* and decreased levels of *Bacteroides*.

*Lactobacillus*-based probiotics have been demonstrated to exert anticoccidial properties on performance parameters such as body weight gain, feed intake, feed conversion ratio, mortality, lesion score and oocysts output [50–52]. They were also shown to have a stimulating effect on the innate and adaptive immune system to stimulate intestinal intraepithelial lymphocyte subpopulations and cytokines including IFN-γ, IL-2, IL-1β and IL-6 [51–53]. To date, limited research has been conducted to study anticoccidial effects of *Lactobacillus*-based probiotics *in vitro*. Tierney et al. [54] determined the inhibition of *E. tenella* sporozoite invasion by *Lactobacillus* species for the first time, whereby three *Lactobacillus* strains and their secreted metabolites in the spent culture supernatant were confirmed to inhibit the parasite invasion into MDBK cells. Hessenberger et al. [18] established a fast and inexpensive *in vitro* tool to screen for probiotics with anticoccidial activity and showed that viable *Lactobacillus reuteri* # 514 and *Lactobacillus salivarius* subsp. *salivarius* # 505 could inhibit parasite invasion by more than 60% at the concentration of 10^7^ CFU/well; however, the spent culture supernatant of both probiotics had no protection on parasite invasion. Considering the increasing resistance of parasites against anticoccidial drugs, *Lactobacillus* based-probiotics should be considered for the control of avian coccidiosis.

Our study focused on deciphering the changes of the gut microbiota composition after *Eimeria* infection and our findings indicate that dysbiosis of some resident flora may contribute to the pathogenicity of caecal coccidiosis. We did not consider the impact of the gut microbiota on coccidia. Therefore, in follow-up studies we plan to screen for *Lactobacillus* strains with anticoccidial activity *in vitro* first and subsequently investigate the potential protective use of suitable *Lactobacillus* strains-based probiotics *in vivo* against *E. tenella*; *Faecalibacterium prausnitzii* is also of interest in this context.

## Conclusions

Altogether, the data in the present study demonstrate that gut microbiota shifts take place during the life-cycle of *E. tenella* infection. Infection with *E. tenella* impacts the cecal microbial composition and structure in AA broiler chickens. Non-pathogenic bacteria such as *Lactobacillus*, *Faecalibacterium*, *Ruminococcaceae* UCG-013, *Romboutsia* and *Shuttleworthia*, decreased in abundance. However, the opportunistic pathogens *Enterococcus* and *Streptococcus* were enriched in abundance. These findings further improve our current understanding of the influence of coccidia on microbiota during infection, indicate a correlation between time-related dysbiosis of gut flora and pathology and provides indications about the optimum time for therapeutic probiotics intervention. Our further research is to investigate the potential probiotics that have anticoccidial effects and may aid in the development of novel control strategies against *E. tenella* infections.

## Data Availability

Data supporting the conclusions of this article are included within the article and its additional files. The raw reads of sequencing results were deposited into the NCBI Sequence Read Archive database under the Accession Number SRP184532.

## Abbreviations

AA broilers: Arbor Acre broilers
hpi.: hours post-infection
FLASH: fast length adjustment of short reads
Qiime: quantitative insights into microbial ecology
OTU: operational taxonomic units
PCA: principal components analysis
LDA: linear discriminant analysis
LEfSe: linear discriminant analysis coupled with the effect size
PCoA: principal coordinates analysis
MDBK: Madin-Darby bovine kidney
